# Supplementary material for: Cold Sintering of Li6.4La3Zr1.4Ta0.6O12/PEO Composite Solid Electrolytes
Source: Molecules. 2022 Oct 10;27(19):6756. doi: 10.3390/molecules27196756 (PMC9572155; doi:10.3390/molecules27196756)
Supplement: Supplementary file 1 [file molecules-27-06756-s001.zip › molecules-1854200-supplementary.pdf]

*Supplementary materials*

# Cold Sintering of $Li_{6.4}La_3Zr_{1.4}Ta_{0.6}O_{12}$/PEO Composite Solid Electrolytes

**Binlang He [1], Shenglin Kang [1], Xuetong Zhao [1,*], Jiexin Zhang [1], Xilin Wang [2], Yang Yang [1], Lijun Yang [1] and Ruijin Liao [1]**

[1] State Key Laboratory of Power Transmission Equipment & System Security and New Technology, Chongqing University, Chongqing 400044, China
[2] Tsinghua Shenzhen International Graduate School, Tsinghua University, Shenzhen 518055, China
* Correspondence: zxt201314@cqu.edu.cn

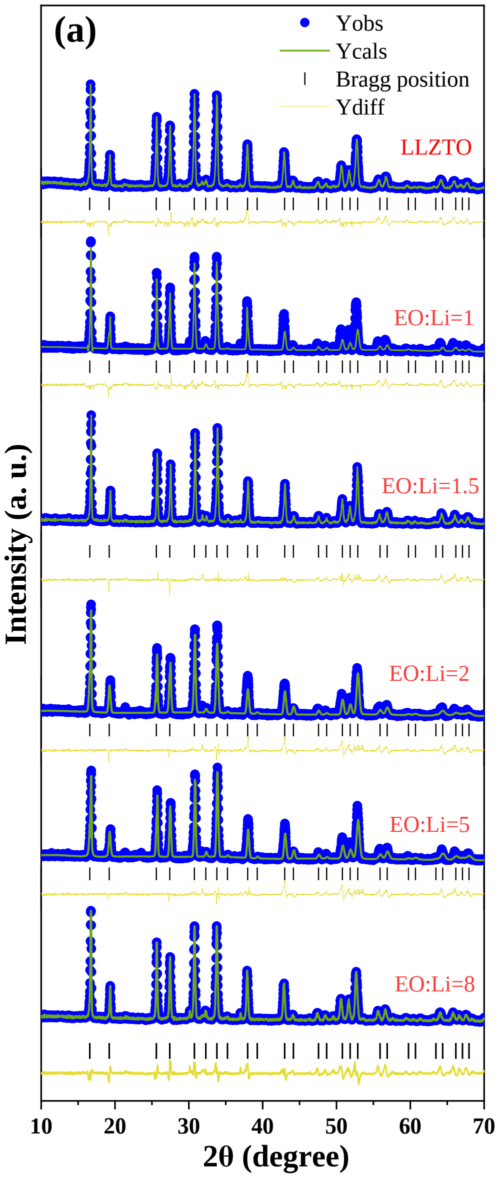


(a)

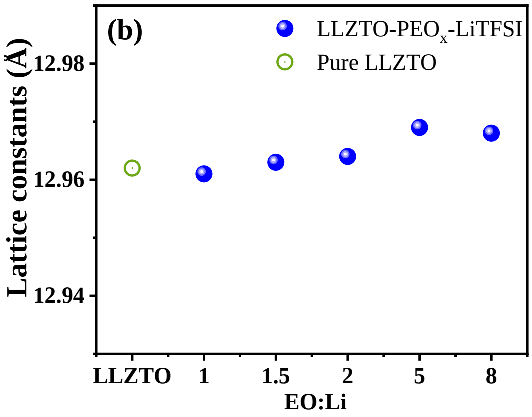


(b)

**Figure S1.** (**a**) Rietveld refinement results of XRD patterns for LLZTO-$PEO_x$-LiTFSI, (**b**) Lattice constants of the cold-sintered LLZTO composite electrolytes.

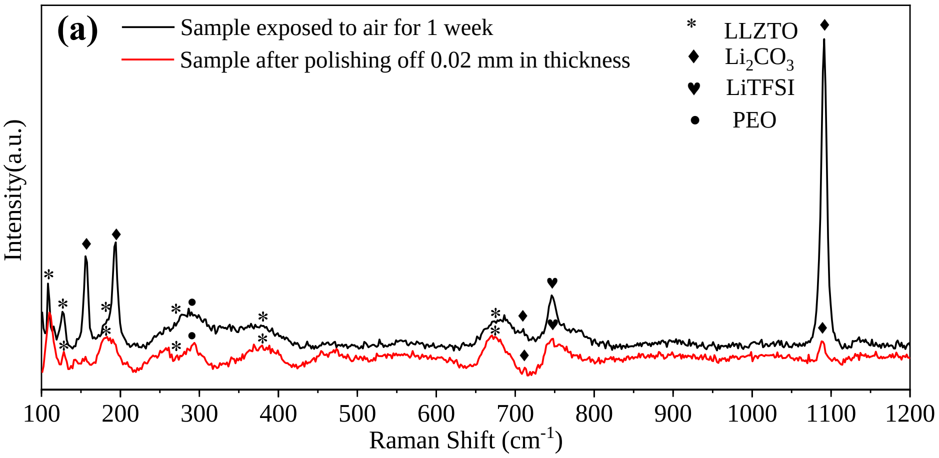


(**a**)

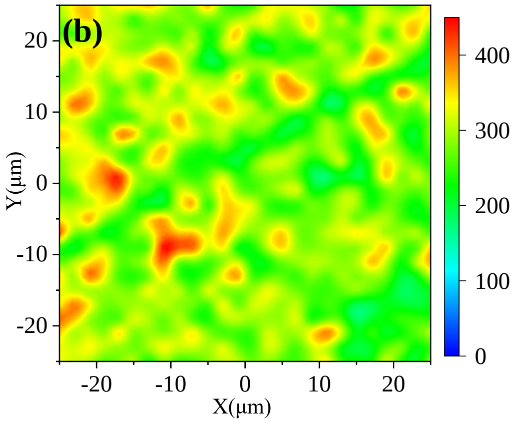


(**b**)

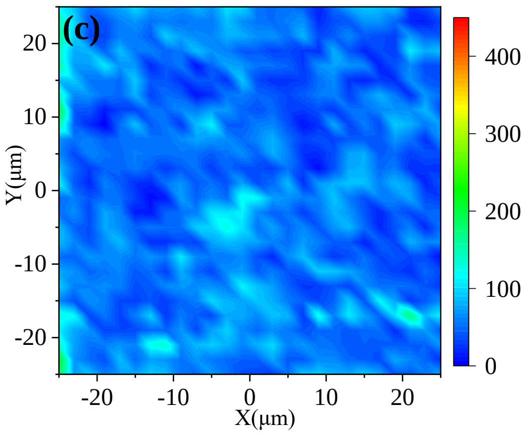


(**c**)

**Figure S2.** (**a**) Typical Raman spectra of the air-exposed LLZTO-PEO2-LiTFSI sample before and after being polished, Raman mapping images of the (**b**) air-exposed LLZTO-PEO2-LiTFSI before being polished and (**c**) after being polished.
